# Cytokine and C-reactive protein concentrations over 6 months of treatment with clozapine in treatment resistant schizophrenia

**DOI:** 10.1016/j.bbih.2026.101342

**Published:** 2026-09-01

**Authors:** Katherine Embleton, Sophie Bennett, James H. MacCabe, Alice Egerton

**Affiliations:** Department of Psychosis Studies, Institute of Psychiatry, Psychology and Neuroscience, King's College London, United Kingdom

## Abstract

Clozapine may have both pro- and anti-inflammatory effects, which could contribute to its unique efficacy in treatment resistant schizophrenia (TRS). Increases in pro-inflammatory cytokines occur during the first weeks after clozapine initiation, but the changes occurring over longer periods of clozapine administration are largely unknown.

This study examined C-reactive protein (CRP), tumour necrosis factor alpha (TNF-α), interferon-gamma (IFN-γ), and interleukins (IL) IL-2, IL-4, IL-6, IL-8, IL-12p70 and IL-13 prior to clozapine titration (baseline) and after 3 and 6 months of clozapine treatment in TRS. Symptom severity was assessed at the same time-points.

Data were available in 53 individuals at baseline, 34 individuals at 3 months and 28 individuals at 6 months. Following correction for false discovery rate (FDR): significant effects of time were observed for IL-10 (F(2, 67.7) = 9.15, P < 0.001, q = 0.010) and TNF-α (F(2, 58.9) = 5.67, P = 0.006; q = 0.030), related to significant increases in both IL-10 and TNF-α at 3 and 6 months after commencing clozapine compared to at baseline (α = 0.05). There were no significant associations between temporal changes in peripheral inflammatory marker concentrations and changes in total symptom severity, or relationships between inflammatory marker concentrations at baseline and total symptom severity at 3 months.

Concurrent increases in TNF-α and IL-10 may reflect increased activation of both pro-inflammatory and compensatory anti-inflammatory mechanisms over 6 months of clozapine treatment. Composite indices of inflammatory profile may be advantageous in future studies examining relationships with symptom improvement during clozapine treatment.

## Highlights

• IL-10 and TNF-α are increased after 3 and 6 months of clozapine treatment in TRS.
• Clozapine may have both pro- and anti-inflammatory effects.
• Cytokines and CRP were not associated with total symptom severity.

## 1 Introduction

There is increasing evidence that inflammatory mechanisms play an important role in the aetiology of schizophrenia (Muller, 2018). Meta-analyses find increases in pro-inflammatory cytokines and C-reactive protein (CRP) in psychosis or schizophrenia compared to in healthy controls (Fernandes et al., 2016; Goldsmith et al., 2016; Pillinger et al., 2019; Upthegrove et al., 2014). Although anti-inflammatory treatments may not be effective for all patients with schizophrenia (Jeppesen et al., 2020), they could show promise in patient subgroups with low-grade inflammation (Foley et al., 2023).

Treatment resistant schizophrenia (TRS) represents a patient subgroup for whom new interventions are urgently needed. Cross-sectional studies indicate an elevated peripheral pro-inflammatory profile in TRS compared to in treatment responsive illness (Chen et al., 2023; Leboyer et al., 2021; Leung et al., 2023). There is also evidence from longitudinal studies linking peripheral inflammatory markers to subsequent clinical outcomes in psychosis. Higher levels of interleukin-6 (IL-6), interleukin-8 (IL-8), interferon gamma (IFN-γ) and CRP, indicating an overall pro-inflammatory profile, associate with worse clinical outcomes in psychosis, whereas higher levels of interleukin-10 (IL-10), generally considered an anti-inflammatory marker, associates with greater symptom improvement (Enache et al., 2021; Kose et al., 2021; Mondelli et al., 2015). While it is challenging to measure neuroinflammation in the human brain *in vivo*, myoinositol, which can be quantified in the brain using proton magnetic resonance spectroscopy, provides a marker of astroglial activation and neuroinflammation (Chang et al., 2013; Rothermundt et al., 2007). We and others have recently reported elevations in frontal cortical myo-inositol in TRS (King et al., 2026; Smucny et al., 2024). Overall, these findings indicate that pro-inflammatory mechanisms may be more active in TRS, which could indicate anti-inflammatory interventions for this subgroup.

Clozapine is the only recommended antipsychotic for TRS but also carries substantial side effect burden. Clozapine may have both pro- and anti-inflammatory effects (Amerio et al., 2024; Giridharan et al., 2020; Maes et al., 2002; Sugino et al., 2009). Cross-sectional studies of peripheral inflammatory markers in TRS have predominantly included patients chronically prescribed clozapine (Chen et al., 2023; Leboyer et al., 2021; Leung et al., 2023). Longitudinal studies are required to separate the effects of clozapine from the effects of TRS illness mechanisms on inflammatory markers. During the first weeks of clozapine initiation, clozapine can induce inflammatory adverse effects including transient fever and eosinophilia. In some cases, these inflammatory effects can extend to more serious events such as myocarditis. Most longitudinal studies of peripheral inflammatory markers have therefore focussed on the initial weeks following clozapine initiation. Studies examining up to 6 weeks of clozapine treatment have reported overall pro-inflammatory effects, including increases in tumour necrosis factor alpha (TNF-α) and IL-6, which may be mainly driven by individuals who develop fever (Hinze-Selch et al., 2000; Kluge et al., 2009; Maes et al., 1997; Pollmacher et al., 1996).

There are few studies of peripheral inflammatory markers over longer periods of clozapine treatment, including in relation to symptomatic response which can continue to emerge from after 6 weeks of treatment (Rosenheck et al., 1999). In 12 individuals examined before and after 10 weeks of clozapine treatment, Monteleone et al., reported a decrease in TNF-α and no change in IL-6 (Monteleone et al., 1997). In 17 individuals examined after 2 and 4 months of clozapine treatment Maes et al., reported no change in IL-6, IL-8 or IL-10, increases in soluble CD8 (sCD8) at 2 months, and increases in leukaemia inhibitory factor receptor (LIF-R) and decreases in Clara Cell protein (CC16) at 4 months, suggesting a mixed pro- and anti-inflammatory effects (Maes et al., 2000, 2002). Although the evidence is limited, together this may suggest that a pro-inflammatory response occurs during the first weeks of clozapine initiation, followed by mixed pro- and anti-inflammatory effects after a longer period of clozapine administration.

The changes in inflammatory profile over longer periods of clozapine administration may be more relevant to the efficacy of clozapine in improving symptoms. Recent cross-sectional studies report elevations in interleukin-2 (IL-2, predominantly pro-inflammatory at higher concentrations), in clozapine-resistant TRS compared to clozapine-responsive TRS (Li et al., 2025), and compared to healthy controls (Dong et al., 2024), and positive correlations between IL-2 and total symptom severity (Li et al., 2025). In contrast, TNF-α is lower in clozapine-resistant TRS compared to clozapine-responsive TRS and negatively correlates with total symptom severity (Li et al., 2025). There is a lack of longitudinal studies of cytokines and CRP in relation to symptom outcomes following clozapine treatment. Higher neutrophil-to-lymphocyte ratio (NLR) predicts longer admission in psychosis (Blackman et al., 2025) and an elevated NLR prior to clozapine initiation predicts symptomatic response to clozapine (Llorca-Bofi et al., 2024). Lastly, reductions in immunoglobulins occurring over 3 months after clozapine initiation correlate with reductions in symptom severity (Griffiths et al., 2024). While cytokine concentrations, NLR and immunoglobulin levels reflect distinct aspects of immune function, together these findings may suggest an overall profile of elevated inflammatory state in TRS, which may predict clinical response to clozapine and resolve as symptoms improve during clozapine treatment.

The primary aim of this study was to investigate changes in cytokine and CRP concentrations after 3 and 6 months of clozapine treatment compared to prior to commencing clozapine, in patients with TRS. The secondary aims were to investigate potential associations between change in concentration of these inflammatory markers with change in symptom severity, and between inflammatory marker concentrations prior to commencing clozapine and subsequent symptom severity at 3 months. Due to the paucity of previous comparable literature, we did not specify directional hypotheses for individual cytokines or CRP, and treated analyses as exploratory.

## 2 Methods

### 2.1 Participants

The study was approved by the London South-East NHS Research Ethics Committee (Ref: 13/LO/1857). Changes in neuroimaging markers, immunoglobulins, and DNA methylation during clozapine treatment have been previously reported in an overlapping cohort (Gillespie et al., 2024; Griffiths et al., 2024; Krajner et al., 2022; McQueen et al., 2021; Sun et al., 2024, 2026). Most participants gave their written informed consent to participate. The study was also open to participants lacking mental capacity to consent, in which instances a consultee advised assent on their behalf. Participants were eligible if they had an ICD-10 diagnosis of schizophrenia (F20) or schizoaffective disorder (F25); were considered treatment resistant, defined as having completed at least two six-week trials of non-clozapine antipsychotics at recommended doses, as inferred from medical records and through discussion with their treating psychiatrist; and were about to commence clozapine as part of their normal clinical care. Exclusion criteria included being under 18 years old, pregnancy, or having been prescribed clozapine in the preceding 3 months. Participants were excluded from subsequent follow-up visits if they did not commence clozapine after the baseline assessment, or discontinued clozapine during the follow-up period.

### 2.2 Clinical measures

Demographic and clinical variables were collected through structured interviews and medical record review at baseline (14 to 0 days prior to clozapine titration). Symptom severity was assessed using Positive and Negative Syndrome Scale for Schizophrenia (PANSS) (Kay et al., 1987), at baseline, 3 and 6 months after commencing clozapine.

### 2.3 Measurement of CRP and cytokine levels

Venous blood samples were collected at baseline, 3 and 6 months after clozapine initiation. Samples were centrifuged, aliquoted for plasma and stored at −80°C. Cytokine and CRP levels (pg/mL) were batch analysed, using MSD Multi-Spot Assay System Proinflammatory Panel 1 (Human)® and MSD U-PLEX Human CRP® kits according to the manufacturer protocols. IL-4 and IL-13 were analysed on a 96-well MULTI-SPOT plate, and IFN-γ, IL-1β, IL-2, IL-6, IL-8, IL-10, IL-12p70, and TNF-α were analysed on individual small spot plates. CRP was tested on a 96-well plate. Samples were diluted 2-fold and distributed across the plates. Each included a seven-point calibration curve with 4-fold dilutions and a zero-calibrator blank. Raw signal values were converted to concentration estimates using the calibration curve, adjusting for dilution.

All measurements were taken in duplicate and the mean value calculated for analyses. Values which were missing due to falling below the curve fit range were imputed using the median lower limits of detection. Values which were returned but flagged as falling below the detection limit were retained to reduce imputation bias. Values missing due to participant dropout were not imputed. IL-1β was excluded from all analyses as over 15% of values were undetected. CRP concentrations were converted from pg/mL to μg/mL for analyses.

### 2.4 Statistical analysis

Statistical analyses were conducted in IBM SPSS Statistics, version 31. Descriptive statistics summarised demographic and clinical characteristics. Longitudinal associations between time on clozapine and inflammatory marker concentrations were assessed using linear mixed-effect models with restricted maximum likelihood estimation (REML). Separate models were fitted for each of the 10 inflammatory markers, with marker concentration as the dependent variable, subject-specific random intercepts, and time as a fixed effect. Models were adjusted for baseline body mass index (BMI), age, and smoking status, which have been associated with cytokine or CRP concentrations (Aldaham et al., 2015; Michaud et al., 2013; Valmorbida et al., 2023). Sex was considered as a potential covariate but was not included in the final analyses as imbalance in sex distribution led to numerical instability in some models. A variance components covariance structure was specified, with participant ID defining repeated observations. Model residuals were visually inspected using Q-Q plots. Where substantial deviations from normality were observed, inflammatory marker concentration values were log-transformed and models re-estimated, with re-inspection of model residuals. In instances where this occurred, as the raw and log-transformed models yielded consistent conclusions, only the log-transformed model is reported. To control the false discovery rate (FDR) across inflammatory markers, model-level P-values were adjusted using the Benjamini and Hochberg procedure. Significant overall effects of time were subsequently investigated using pairwise comparisons between time-points, using estimated marginal means. For significant results, subsequent sensitivity analyses determined whether these effects remained when analysis was restricted to participants in whom data were available at all timepoints; were clozapine-naïve at baseline; without physical health conditions; or not prescribed non-CNS medications.

Longitudinal associations between changes in inflammatory marker concentrations and total symptom severity were also examined using linear mixed-effects models estimated by REML. PANSS total score was specified as the dependent variable, time-point was specified as a fixed effect and participant ID as a random intercept. Inflammatory marker concentrations were decomposed into within-individual components, by calculating the participant mean concentration across time points and the difference between the observed concentration at each time-point and the participant's mean. This within-participant variable was entered as a time-varying covariate and constituted the primary predictor of interest. As time-invariant characteristics do not confound within-person associations, these models did not include BMI, smoking or age. Separate models were fitted for each inflammatory marker. Model residuals were visually inspected using Q–Q plots to assess normality.

Associations between inflammatory markers at baseline and PANSS total symptom severity at 3 months were evaluated using linear regression, adjusting for baseline PANSS total symptom severity score. Model residuals were visually inspected using Q–Q plots.

## 3 Results

### 3.1 Demographic and clinical characteristics

Data were available in 53 participants at baseline (38 male, mean ± standard deviation (SD) age = 39.06 ± 12.98 years; BMI = 29.86 ± 6.53; duration of illness = 12.71 ± 9.37 years). Of the baseline sample, 8 had previously been exposed to clozapine. Smoking status included none (n = 33); daily (n = 18); and occasional or unknown (n = 2). Of the baseline sample, 5 participants reported one or more physical health conditions, including type II diabetes mellitus, obesity, hypertension, hyperthyroidism and Raynaud's disease. Non-CNS medications included metformin (4 participants), and in 3 participants one or more of salbutamol, budesonide plus formoterol (Symbicort), fexofidine or co-codamol.

Follow-up data was available in 34 participants at 12 weeks and 28 participants at 24 weeks. 26 participants completed all 3 study visits. Attrition reasons included declining study blood draws or broader disengagement from study participation (n = 14), not commencing clozapine treatment (n = 2), discontinuing clozapine either due to non-adherence or due to side effects, which included sedation, low neutrophil counts or cardiac concerns (n = 9). PANSS total symptom scores improved from mean ± SD 73.10 ± 17.10 at baseline, to 59.22 ± 17.29 at 3 months, to 56.96 ± 17.26 at 6 months. The mean clozapine dose at 3 months was 323.08 ± 152.30 mg/day and at 6 months 347.50 ± 164.21 mg/day. Plasma levels of clozapine and nor-clozapine at 3 months were: n = 26, clozapine: 0.45 ± 0.25 mg/L, range: 0.21 to 1.52 mg/L; nor-clozapine: 0.61 ± 0.26 mg/L, range 0.10 to 0.61 mg/L; and at 6 months, n = 14, clozapine: 0.44 ± 0.12 mg/L, range 0.29 to 0.69 mg/L, nor-clozapine 0.25 ± 0.10 mg/L, range 0.15 to 0.45 mg/L, however levels were not measured at a standardised time relative to clozapine dosing.

### 3.2 Longitudinal effects of clozapine treatment on peripheral inflammatory marker concentrations

**Fig. 1:**
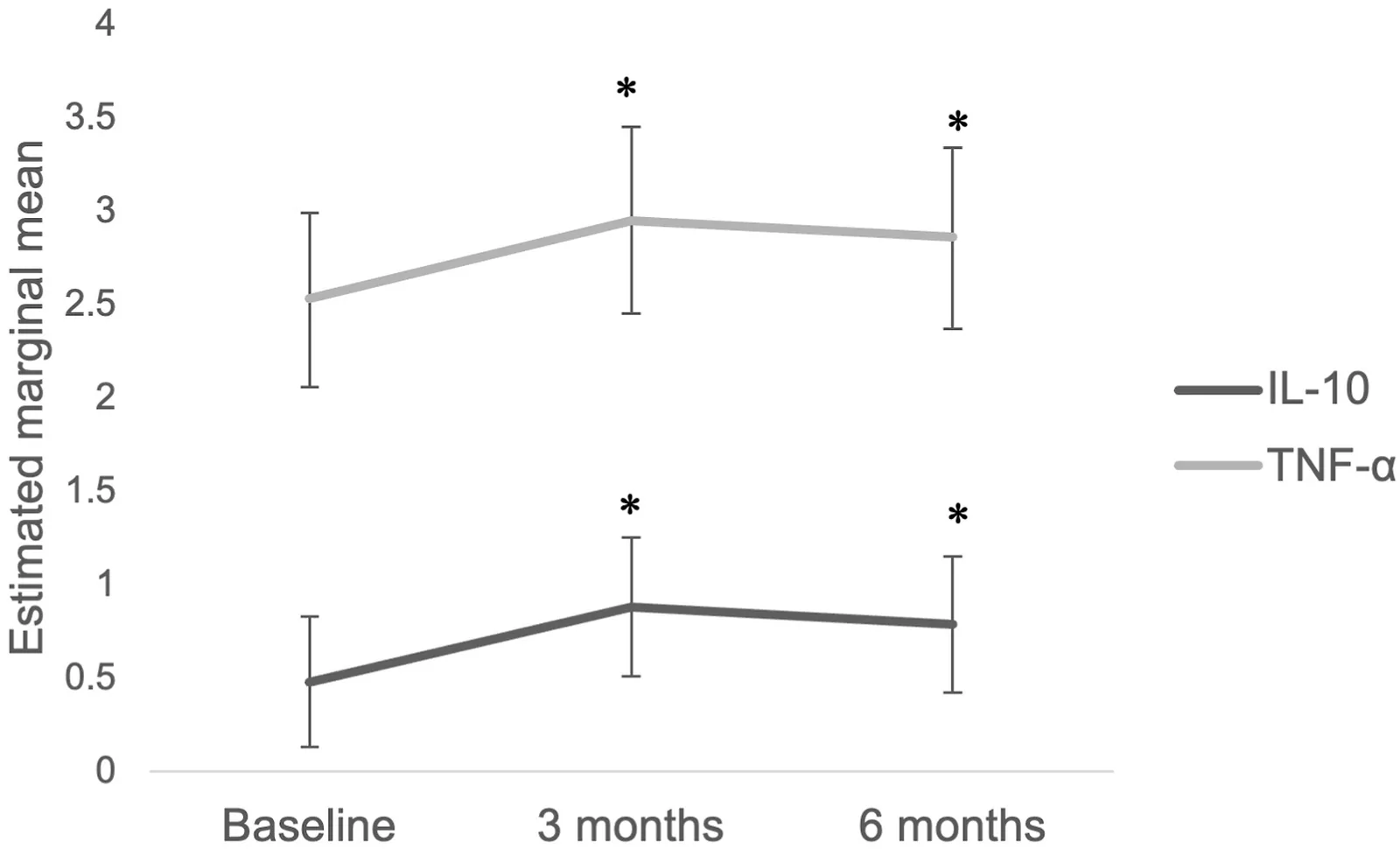
Estimated Marginal Means for interleukin-10 (IL-10) and tumour necrosis factor alpha (TNF-α) prior to commencing clozapine (Baseline, n = 53) and after 3 (n = 34) and 6 (n = 28) months of clozapine treatment. Asterisks indicate significant pairwise comparisons compared to baseline. Error bars represent 95% confidence intervals.

Significant effects of time were observed for IL-10, (F_(2, 67.7)_ = 9.15, P < 0.001), TNF-α (F_(2, 58.9)_ = 5.67, P = 0.006) and IL-6 (F_(2, 54.9)_ = 3.47, P = 0.038). There was no evidence of a time effect for CRP, IFN-γ, IL-2, IL-4, IL-8, IL-12p70 or IL-13 (all P > 0.05, Table 1). After FDR-correction, the effect of time remained significant for IL-10 (q = 0.010) and TNF-α (q = 0.030), but not IL-6 (q = 0.130). The estimated marginal means for each inflammatory marker at each time point are presented in Table 2. Pairwise comparisons showed a significant increase of IL-10 from baseline to 3 months (mean difference = 0.399, SE = 0.099, df = 70.4, P < 0.001, 95% CI [0.201, 0.597]) and from baseline to 6 months (mean difference = 0.315, SE = 0.107, df = 68.9, P = 0.005, 95% CI [0.101, 0.529], but no significant change between 3 and 6 months (mean difference = 0.084, P = 0.463, 95% CI [−0.144, 0.312]) (Fig. 1). Pairwise comparisons also showed a significant increase in TNF-α from baseline to 3 months (mean difference = 0.422, SE = 0.134, df = 58.8, P = 0.002, 95% CI [0.153, 0.691]) and from baseline to 6 months (mean difference = 0.330, SE = 0.145, df = 58.8, P = 0.025, 95% CI [0.040, 0.620]), but no significant difference between 3 and 6 months (mean difference = 0.092, df = 58.8 P = 0.550, 95% CI [−0.208, 0.392]) (Fig. 1).

**Table 1:** Results of linear mixed-effects models examining fixed effect of time since initiating clozapine (baseline, 3 months, 6 months) on inflammatory marker concentrations, adjusted for baseline BMI (evaluated at = 29.49), age (evaluated at = 39.12) and smoking status. CRP: C-reactive protein, IFN: interferon; IL: interleukin; TNF: tumour necrosis factor.

Marker	Time F _(df)_	P value	FDR-adjusted q
Log CRP	F_(2, 60.3)_ = 1.99	0.146	0.365
Log IFN-γ	F_(2, 66.5)_ = 0.35	0.706	0.706
IL-2	F_(2, 50.6)_ = 1.00	0.375	0.536
Log IL-4	F_(2, 70.8)_ = 0.77	0.466	0.583
IL-6	F_(2, 54.9)_ = 3.47	0.038	0.127
Log IL-8	F_(2, 69.8)_ = 0.53	0.591	0.657
IL-10	F_(2, 67.7)_ = 9.15	<0.001	**0.010**
IL-12p70	F_(2, 60.6)_ = 1.22	0.304	0.507
IL-13	F_(2, 69.1)_ = 0.82	0.444	0.555
TNF-α	F_(2, 58.9)_ = 5.67	0.006	**0.030**

**Table 2:** Estimated marginal means derived from linear mixed-effects models including random intercepts for participants and fixed effects of time, adjusted for baseline BMI, age and smoking status. CRP: C-reactive protein, IFN: interferon; IL: interleukin; TNF: tumour necrosis factor.

Marker	Baseline (n = 53) Mean (SE [95% CI])	3 months (n = 34) Mean, SE, [95% CI]	6 months (n = 28) Mean, SE, [95% CI]
Log CRP	1.91 (0.32 [1.27, 2.56])	2.28 (0.35 [1.58, 2.98])	2.22 (0.34 [1.54, 2.90])
Log IFN-γ	1.81 (0.18 [1.45, 2.17])	1.91 (0.20 [1.51, 2.31])	1.84 (0.20 [1.45, 2.23])
IL-2	0.46 (0.09 [0.27, 0.65)]	0.46 (0.10 [0.25, 0.67])	0.38 (0.10 [0.18, 0.57])
Log IL-4	−2.83 (0.165 [-3.15, −2.50])	−2.87 (0.19 [-3.24, −2.50])	−3.00 (0.18 [-3.36, −2.63])
IL-6	1.78 (0.38 [1.01, 2.55])	2.18 (0.40 [1.38, 2.98])	1.87 (0.39 [1.08, 2.66])
Log IL-8	1.55 (0.16 [1.24, 1.86])	1.63 (0.17 [1.28, 1.98])	1.51 (0.17 [1.18, 1.85])
IL-10	0.48 (0.18 [0.13, 0.82])	0.88 (0.19 [0.50, 1.25])	0.79 (0.18 [0.44, 1.16])
IL-12p70	0.39 (0.10 [0.19, 0.58])	0.32 (0.10 [0.12, 0.53])	0.34 (0.10 [0.14, 0.54])
IL-13	2.04 (0.35 [1.34, 2.74])	1.89 (0.39 [1.11, 2.67])	1.70 (0.38 [0.94, 2.46])
TNF-α	2.53 (0.23 [2.06, 2.99])	2.95 (0.25 [2.45, 3.45])	2.86 (0.25 [2.37, 3.34])

In sensitivity analysis, the main findings for IL-10 and TNF-α remained significant when including only the 26 participants in whom data were available at all 3 timepoints (main effects of time IL-10: F_(2, 45.3)_ = 7.15, P = 0.002; TNF-α: F_(2, 45.0)_ = 7.15, P = 0.002); only participants who were clozapine naïve at baseline (IL-10: F_(2, 54.1)_ = 8.25, P < 0.001; TNF-α: F_(2, 46.1)_ = 6.38, P = 0.004); only participants without physical health conditions (IL-10: F_(2, 61.9)_ = 9.50, P < 0.001; TNF-α: F_(2, 50.5)_ = 5.70, P = 0.006) or only participants not prescribed non-CNS medications (IL-10: F_(2, 59.0)_ = 8.45, P < 0.001; TNF-α: F_(2, 50.3)_ = 4.51, P = 0.016). In all cases, the associated pairwise comparisons again indicated increases in IL-10 and TNF-α at 3 and 6 months compared to baseline, but no change between 3 and 6 months.

### 3.3 Associations between within-individual fluctuations in inflammatory markers and symptom severity

Within-person fluctuation in IL-6 showed a nominal association with fluctuation in symptom severity (P = 0.043, Table 3), such that participants tended to report more severe symptoms when IL-6 was elevated relative to their own average. This effect was not significant after FDR correction (q = 0.430). Within-person fluctuations in the remaining inflammatory markers were not significantly associated with PANSS total scores over the observation period (Table 3).

**Table 3:** β coefficients represent within-person effects estimated from linear mixed-effects models with random intercepts for participants and adjustment for time. CRP: C-reactive protein, IFN: interferon; IL: interleukin; TNF: tumour necrosis factor.

Marker	Β	SE	df	T	P-value	95% CI
CRP	0.024	0.137	56.8	0.18	0.861	−0.250, 0.299
IFN-γ	−0.070	0.184	58.4	−0.38	0.707	−0.438, 0.299
IL-2	5.510	5.162	56.8	1.07	0.290	−4.828, 15.847
IL-4	49.896	33.082	56.8	1.51	0.137	−16.353, 116.145
IL-6	4.116	1.984	56.6	2.08	0.043	−0.143, 8.088
IL-8	0.209	0.573	57.9	0.37	0.716	−0.938, 1.356
IL-10	0.606	2.990	57.8	0.20	0.840	−5.380, 6.592
IL-12p70	6.050	6.652	56.9	0.91	0.367	−7.271, 19.371
IL-13	1.028	1.127	55.9	0.91	0.365	−1.228, 3.284
TNF-α	2.470	2.231	58.2	1.11	0.273	−1.996, 6.936

### 3.4 Associations between baseline inflammatory markers concentration and symptom severity at 3 months

Baseline CRP showed a nominal positive association with symptom severity at 3 months (P = 0.034, Table 4). This effect was not significant after FDR correction (q = 0.340). The remaining inflammatory markers showed no associations with PANSS total scores at the 3 month follow-up.

**Table 4:** β coefficients for effect of baseline inflammatory markers on PANSS total symptom severity score at 3 months, estimated from linear regression covarying for PANSS total score at baseline. CRP: C-reactive protein, IFN: interferon; IL: interleukin; TNF: tumour necrosis factor.

Marker	Β	SE	t	P-value
CRP	0.080	0.036	2.22	0.034
IFN-γ	0.310	0.462	0.67	0.508
IL-2	3.475	7.150	0.49	0.630
IL-4	−54.160	−52.862	−1.03	0.313
IL-6	0.335	1.860	0.18	0.858
IL-8	−1.015	0.960	−1.06	0.298
IL-10	−10.740	6.006	−1.79	0.083
IL-12p70	−7.821	5.176	−1.51	0.140
IL-13	−0.197	1.445	−0.14	0.892
TNF-α	1.763	3.032	0.58	0.565

## 4 Discussion

This study examined peripheral cytokine and CRP concentrations prior to commencing clozapine and after 3 and 6 months of clozapine treatment in TRS. The main finding was that TNF-α and IL-10 were increased at 3 and 6 months compared to at baseline (prior to clozapine titration). There was a trend for an increase in IL-6 over time, which did not survive FDR correction. No effects of time on CRP, IFN-γ, IL-2, IL-4, IL-8, IL-12p70 or IL-13 were detected.

Our data indicate that increases in TNF-α occurring 6 weeks into clozapine treatment (Hinze-Selch et al., 2000; Kluge et al., 2009; Pollmacher et al., 1996) may be sustained up to 6 months into treatment. TNF-α is a central pro-inflammatory cytokine rapidly produced following immune activation. IL-10 can suppress production of TNF-α and other proinflammatory mediators, thereby limiting inflammatory responses (Moore et al., 2001; Smallie et al., 2010). The increases in both TNF-α and IL-10 observed after clozapine treatment may therefore reflect increased inflammation and activation of regulatory anti-inflammatory mechanisms. This interpretation would potentially be consistent with the inflammatory response system (IRS) and compensatory immune-regulatory reflex system (CIRS) framework in schizophrenia, where immune activation is accompanied by attenuating counter-regulatory mechanisms (Roomruangwong et al., 2020). Compared to non-TRS, TRS may be associated with a shift in the IRS/CIRS equilibrium towards an increased pro-inflammatory profile, associated with greater symptom severity and lateral ventricle enlargement (Chen et al., 2023). It would be of interest to conduct a similar analysis of potential changes in IRS/CIRS balance during clozapine treatment.

Although below the threshold for statistical significance after FDR correction, there was also a trend for an increase in IL-6 over the observation period. IL-6 is a pro-inflammatory marker which also increases during the first weeks of clozapine treatment (Kluge et al., 2009; Maes et al., 1997; Pollmacher et al., 1996). Increases in TNF-α, IL-10 and IL-6 after clozapine treatment would be broadly consistent with cross-sectional elevations in these cytokines in individuals with TRS treated with clozapine compared to control (Chen et al., 2023; Fang et al., 2020; Maes et al., 2002; Yuan et al., 2022), and with positive correlations between these markers in clozapine-treated TRS (Krivoy et al., 2020).

Within-participant fluctuations in TNF-α and IL-10 over the observation period were not significantly associated with fluctuations in total symptom severity, and concentrations of TNF-α and IL-10 prior to clozapine titration were not predictive of total symptom severity after 3 months of clozapine treatment. There was a nominal association between within-person fluctuation in IL-6 and within-person fluctuation in total symptom severity, such that when IL-6 was higher total symptom severity also tended to be higher, which did not survive FDR correction. Potentially, composite indices of inflammation, such as the IRS/CIRS ratio (Roomruangwong et al., 2020) may be more strongly associated with clozapine response. Such composite indices may also relate more closely to the neutrocyte to lymphocyte ratio (NLR), recently associated with clozapine response (Llorca-Bofi et al., 2024), which reflects downstream cellular consequences of inflammatory balance.

Emerging research in schizophrenia finds that peripheral inflammatory cytokines associate with brain structural features (Chen et al., 2023), functional markers (Lizano et al., 2023; Mlynek et al., 2026) and metabolite concentrations (Deakin et al., 2026; Fenn-Moltu et al., 2023). Changes in cortical thickness and subcortical volume (Ahmed et al., 2015; Krajner et al., 2022; Tronchin et al., 2020), striatal microstructure (Jo et al., 2026; Raminfard et al., 2026; Tronchin et al., 2021), metabolites including glutamate (McQueen et al., 2021), cerebral blood flow (Sun et al., 2024, 2026), cortico-striatal connectivity (Blazer et al., 2022) also occur during clozapine treatment. Future longitudinal studies could examine associations between changes in peripheral inflammatory markers and brain neurobiological features in relation to clozapine response in TRS.

Strengths of our study include the prospective longitudinal design, conducted in a larger cohort and examining a longer period of clozapine administration compared to previous studies (Hinze-Selch et al., 2000; Kluge et al., 2009; Maes et al., 1997, 2000, 2002; Monteleone et al., 1997; Pollmacher et al., 1996). Limitations include attrition during the follow-up period, which in some cases was due to discontinuing clozapine due to adverse effects which could be related to inflammatory mechanisms. While it is therefore possible that greater changes in inflammatory markers may have occurred in some individuals who withdrew, increases in TNF-α and IL-10 remained significant in sensitivity analysis restricted to participants with data at all timepoints. To determine specificity to clozapine, the longitudinal changes in inflammatory marker concentrations in TRS must be compared to those occurring following treatment with non-clozapine antipsychotics (Casetta et al., 2024), however clozapine is the only recommended antipsychotic for adults with TRS. There is some evidence that inflammatory signatures in schizophrenia may particularly relate to negative symptoms or cognitive performance (Dunleavy et al., 2022; Lizano et al., 2023). Due to the exploratory nature of the study, we restricted analyses to examining relationships with total symptom severity, and our study did not acquire cognitive measures. Although our analysis adjusted for baseline BMI and smoking status, we did not account for changes in weight, smoking behaviour or other time-varying covariates during clozapine treatment which could potentially impact inflammatory markers.

In conclusion, increases in TNF-α and interleukin IL-10 are apparent after 3 and 6 months of clozapine treatment in TRS compared to prior to clozapine initiation, indicating activation of both pro-inflammatory and anti-inflammatory mechanisms. Future studies could examine the early and prolonged effects of clozapine on composite panels of inflammatory markers and investigate their associations with brain structural or functional features.

## CRediT authorship contribution statement

**Katherine Embleton:** Data curation, Formal analysis, Writing – original draft, Writing – review & editing. **Sophie Bennett:** Data curation, Writing – review & editing. **James H. MacCabe:** Conceptualization, Funding acquisition, Investigation, Methodology, Supervision, Writing – review & editing. **Alice Egerton:** Conceptualization, Data curation, Formal analysis, Funding acquisition, Investigation, Methodology, Project administration, Resources, Supervision, Writing – original draft.

## Ethics declaration

Written informed consent to take part in the study and to publish the article has been obtained from all participants or their legal representatives. The privacy rights of participants have been observed.

This study included organ or tissue donors. This study includes human biological material and consent was obtained by donors, or their next of kin or legal representatives, for use in this study and for publication of the article. The samples used in this research were not sourced from executed prisoners or prisoners of conscience.

This study was performed in compliance with relevant laws, regulatory frameworks and guidelines where the research took place. This study was approved by the London South-East NHS Research Ethics Committee. (Approval No. 13/LO/1857)

## Disclosures

A.E. has received consultancy fees from Leal Therapeutics Ltd and Newron Pharmaceuticals.

J.H.M. has received investigator-initiated research funding from H Lundbeck. He has received research funding for clinical trials from H Lundbeck, Bristol Myers Squibb and Newron Pharmaceuticals. He has participated in advisory boards for H Lundbeck, Bristol Myers Squibb, LB Pharma, Newron Pharmaceuticals and Teva UK Ltd.

## Funding

The study was supported by the 10.13039/501100000265Medical Research Council, UK, Grant 10.13039/100012573MR/L003988/1 to A.E. and by the European Community's Seventh Framework Programme (FP7/2007–2013), grant 279227 to J.H.M. This study presents independent research funded in part by the 10.13039/501100000272National Institute for Health and Care Research (10.13039/501100000272NIHR), Biomedical Research Centre: Maudsley, South London and Maudsley National 10.13039/100018696Health Service (10.13039/100030827NHS) Foundation Trust, London, United Kingdom.

## Declaration of competing interest

The authors declare the following financial interests/personal relationships which may be considered as potential competing interests: Alice Egerton reports financial support was provided by UK Research and Innovation Medical Research Council. James H MacCabe reports financial support was provided by European Commission Seventh Framework Programme for Research and Technological Development Health. Alice Egerton reports financial support was provided by NIHR Maudsley Biomedical Research Centre. Alice Egerton reports a relationship with Leal Therapeutics Inc that includes: consulting or advisory. Alice Egerton reports a relationship with Newron Pharmaceuticals SpA that includes: speaking and lecture fees. James MacCabe reports a relationship with Bristol-Myers Squibb Company that includes: consulting or advisory and funding grants. James MacCabe reports a relationship with Newron Pharmaceuticals SpA that includes: consulting or advisory and funding grants. James H MacCabe reports a relationship with LB Pharma that includes: consulting or advisory. James H MacCabe reports a relationship with Teva UK Ltd that includes: consulting or advisory. James H MacCabe reports a relationship with Lundbeck LLC that includes: consulting or advisory and funding grants. If there are other authors, they declare that they have no known competing financial interests or personal relationships that could have appeared to influence the work reported in this paper.
